# HGF Aggravated Periodontitis-Associated Gut Barrier and Microbial Dysfunction: Implications for Oral–Gut Axis Regulation

**DOI:** 10.3390/biology14050496

**Published:** 2025-05-02

**Authors:** Zhen Chen, Yang Zhong, Lu Chen, Weijia Liu, Chuyin Lin, Yannan Chen, Xinhong Wang

**Affiliations:** 1 School of Stomatology, Guangzhou Medical University, Guangzhou 510180, China; zhen_chen2022@163.com (Z.C.); shouko233@163.com (Y.Z.); drchenlu2025@163.com (L.C.); lliuwjj96@163.com (W.L.); llincy21@163.com (C.L.); chenyannan0206@163.com (Y.C.); 2 Guangdong Engineering Research Center of Oral Restoration and Reconstruction, Guangzhou Key Laboratory of Basic and Applied Research of Oral Regenerative Medicine, Guangzhou Medical University, Guangzhou 510180, China

**Keywords:** periodontitis, hepatocyte growth factor, intestinal barrier, gut microbiota, oral–gut axis

## Abstract

While periodontitis is increasingly linked to systemic disorders through the oral–gut axis, the molecular mediators driving gut microbiota dysbiosis and barrier disruption remain elusive. Hepatocyte growth factor (HGF), a novel regulator of inflammatory bone loss in periodontitis, may serve as a critical communicator between oral infection and distal intestinal pathology. This study investigates how HGF overexpression modulates the gut microbial ecosystem and intestinal barrier integrity in a transgenic periodontitis model. In this study, we combined 16S rRNA sequencing of fecal microbiota with comprehensive gut barrier assessments, including systemic markers (D-lactate, LPS, and DAO ELISA), structural integrity (villous morphology), and molecular analysis (ZO-1, occludin, and NOD2 immunohistochemistry), using HGF-overexpressing transgenic (HGF-Tg) mice with periodontitis. The results demonstrated that HGF increased gut permeability in the context of periodontitis, as evidenced by elevated serum levels of D-lactate and LPS compared to wild type (WT) mice. In addition, gut villous morphology disorder was observed in HGF-Tg mice with periodontitis. HGF also diminished the protein level of occludin and upregulated NOD2 expression in mice with periodontitis. Moreover, HGF-Tg mice with periodontitis exhibited significant dysbiosis of gut microbiota, with reduced levels of probiotics (e.g., *Faecalibaculum*). Notably, HGF also increased the enrichment of the periodontitis-associated pathogens (e.g., *Desulfovibrio* and *Streptococcus*) in the gut. Microbial functions, particularly metabolic pathways, were significantly altered by HGF when periodontitis occurred. Some microorganisms like *g_Desulfovibrio* may play a role in gut barrier disorder in HGF-Tg mice with periodontitis. Overall, our findings position HGF as a novel orchestrator of oral–gut crosstalk, where its overexpression reshapes gut microbial ecology toward a “leaky gut” phenotype to compromise intestinal barrier integrity, further deepening our understanding of the oral–gut axis.

## Simple Summary

Periodontitis, a common oral inflammatory disease, has been increasingly linked to systemic health issues through the oral–gut axis. However, the key molecules contributing to gut microbiota imbalance and intestinal barrier dysfunction in this process remain unclear. Hepatocyte growth factor (HGF) is a protein that regulates inflammation and bone loss. This study aims to explore its role in gut health during periodontitis. Using a mouse model genetically modified to overproduce HGF, we found that high levels of HGF worsened the gut barrier by damaging proteins that hold intestinal cells together, increasing harmful bacteria and reducing beneficial bacteria. These changes made the gut more permeable, allowing toxins to enter the bloodstream. Our findings suggest that HGF plays a crucial role in connecting oral inflammation to gut health disturbances, potentially contributing to broader systemic complications. Understanding this connection may help develop new therapeutic strategies for periodontitis-related diseases.

## 1. Introduction

Periodontitis, a chronic infectious disease triggered by dysbiotic oral microbiota, is characterized by the progressive destruction of periodontal tissues and systemic inflammation [1]. Notably, the intestinal barrier, a critical guardian of gut homeostasis comprising mechanical, immunological, and microbial components, is vulnerable to periodontitis-induced damage [2]. Firstly, periodontitis leads to the downregulation of tight junction proteins (e.g., ZO-1 and occludin) and increases gut permeability to facilitate mechanical barrier impairment [3]. Periodontitis also elevates gut mucosal cytokines (IL-6, IL-1β, and TNF-α) and Th17 cell infiltration to create a pro-inflammatory environment, thereby exacerbating immune barrier damage [4,5]. Additionally, the influx of oral pathobionts may interact with resident gut microbes to cause microbial imbalance, further amplifying intestinal and systemic inflammation [6,7]. These disruptions contribute to a “leaky gut” phenotype, allowing gut-derived pathogens and metabolites to enter circulation, potentially linking periodontitis to extraoral comorbidities [8].

Consequently, the concept of the oral–gut axis, a pathway where periodontal pathobionts perturb intestinal homeostasis, has recently gained traction [9]. This paradigm posits that orally derived pathogens (e.g., *Streptococcus* and *Porphyromonas. gingivalis*) can translocate to the distal intestine, reshaping microbial communities and compromising gut barrier integrity [6,7,10]. This microbial crosstalk is further evidenced by a study where saliva transplantation from periodontitis patients induced gut dysbiosis in mice, suggesting that oral-derived microbes seed intestinal ecological imbalances [9].

While microbial translocation dominates the current oral–gut axis research, it remains unclear whether host-derived mediators exacerbate these microbial-translocation-induced gut injuries and microbial disorders. Hepatocyte growth factor (HGF), a pleiotropic cytokine, exerts wide-ranging effects on epithelial and endothelial cell proliferation, morphogenesis, cancer progression, and inflammatory response [11,12,13]. Accumulating evidence indicates that HGF can influence the activation of immune cells, modulate cytokine production, and regulate signaling molecules such as NF-κB, thereby participating in the progression of inflammatory disorders [14,15]. Clinical studies have reported elevated levels of HGF in the gingival crevicular fluid (GCF) of patients with periodontitis, where its concentration correlates with disease severity [16,17]. Furthermore, the protein level of HGF was markedly reduced after non-surgical periodontal therapy, suggesting that HGF expression dynamically responds to local inflammatory status [16,17]. In our previous study, we found that HGF was upregulated in mice with periodontitis by aggravating the progress of periodontitis via the IL-17/RANKL/TRAF6 pathway using HGF high-expression transgenic (HGF-Tg) mice [12]. These results indicate that HGF is a potential candidate in bridging local periodontal inflammation and distant gut dysfunction.

Beyond its role in periodontitis, HGF is a double-edged sword in intestinal structure and function. On the one hand, in vitro experiments have demonstrated that macrophages can promote intestinal epithelial cell repair by secreting HGF [18]. In one animal model of intestinal burns, intestinal infusion of HGF promoted proliferation and inhibited the apoptosis of intestinal epithelial cells [19]. On the other hand, HGF is dramatically upregulated in the serum and intestinal mucosa of patients with colitis [20]. Reducing the expression of c-Met (the receptor of HGF) in colitis neutrophils alleviates the severity of colitis [20], indicating that HGF may be involved in gut damage. However, no studies have investigated whether HGF exacerbates periodontitis-induced gut dysbiosis—a critical gap in understanding oral–gut axis pathogenesis.

To address the gap, we employed HGF-Tg mice with ligature-induced periodontitis to investigate the overlooked role of HGF in modulating gut ecology and barrier function. Specifically, we aimed to decipher how HGF overexpression altered gut microbiota composition (with a focus on periodontitis-related pathobionts) and assess gut barrier compromise at mechanical, immunological, and functional levels. Finally, we established correlations between HGF-associated dysbiosis and barrier impairment. This study positions HGF as a host factor that may potentiate oral pathogen fitness in the gut—a novel candidate bridging periodontal infection and systemic comorbidities.

## 2. Materials and Methods

### 2.1. Animals and Periodontitis Model

C57BL/6 wild-type mice (WT) were obtained from the Guangdong Experimental Animal Center. The HGF high-expression transgenic mice (HGF-Tg, C57BL/6J background) were generated with previously established protocols [21]. Successful high expression of HGF was verified by PCR amplification of DNA isolated from mice intestine using previously described primers (Figure S1) [21]. Each cage housed five mice in a specific pathogen-free environment with a 12/12 h light–dark cycle at 20–24 °C and a humidity of 50–60%, providing free access to water and food. After a three-day period of quarantine and acclimatization, the mice aged six weeks were randomly divided into four groups (*n* = 6 per group): WT mice (C), HGF-Tg mice (H), WT mice with periodontitis (P), and HGF-Tg mice with periodontitis (HP). Sample size estimation was predicated on an initial experiment. The primary outcomes assessed were bone volume/total volume of periodontal alveolar bone (BV/TV) and serum level of D-lactate. Utilizing software (v3.1, G*power), the analysis determined a requisite sample size of 6 for each group to achieve an 80% power level and a 5% significance level (two-sided) [22]. Experimental periodontitis was induced using a ligature procedure after anesthetizing by pentobarbital (40 mg/kg) through intraperitoneal injection. Then, 5-0 sterile silk sutures were subgingivally ligatured around both maxillary second molar teeth for four weeks [12]. No ligatures were lost during the study, and the alveolar bone loss was assessed and published in our previous study [12]. After 4 weeks, all mice were euthanized by cervical dislocation following intraperitoneal injection of pentobarbital (40 mg/kg) [23]. The Guangdong Huawei Testing Co., Ltd.’s Animal Research Ethics Committee (Shenzhen, Guangdong, China) granted approval for all animal care and experimental protocols (Approval No. HWT-BG-117).

### 2.2. Histologic Analyses and Immunohistochemistry Staining

Ileum tissue samples were preserved in a solution containing 4% buffered formalin for a duration of 24 h. After this, the samples were encased in paraffin, cut into 4 μm sections, and subsequently subjected to hematoxylin and eosin (HE) staining. Intestinal villus height (IVH), intestinal crypt depth (ICD), and the ratio of villus height to crypt depth (H/D) were assessed using Image-Pro Plus by Xinhong Wang and Lu Chen with a Cohen’s Kappa coefficient exceeding 0.85 [24]. IVH is measured from the tip of the intestinal villus to the mouth of the corresponding intestinal crypt, while ICD is measured from the mouth of the intestinal crypt to its base [24].

After deparaffinization by dimethylbenzene, antigen recovery was performed using a citrate antigen retrieval solution (pH = 6, Beyotime, Shanghai, China) at 100 °C for 10 min. Then, endogenous peroxidases were blocked by 3% H_2_O_2_ for 10 min. Next, QuickBlock™ blocking buffer (P0260, Beyotime, Shanghai China) was added, and anti-Occludin (cat: 27260-1-AP, Proteintech, Wuhan, China), anti-ZO-1 (cat: bs-1329R, Bioss, Beijing, China), and anti-NOD2 antibodies (cat: 20980-1-AP, Proteintech, Wuhan, China) were used for incubating with the ileum sections overnight at 4 °C. Then, secondary antibody (abs50012, Absin, Shanghai, China) and DAB (SignalStain^®^ DAB Substrate Kit, CST, Danvers, MA, USA) were added to the paraffin slides in turn. Finally, the nuclei were counterstained with hematoxylin. Mean density (MOD) and integrated density (IOD) were measured to reflect the expression levels of proteins using Image-Pro Plus [25].

### 2.3. Intestinal Permeability Evaluation

Peripheral blood was collected and centrifugated to obtain serum. The serum levels of LPS (cat.no. CSB-E13066m, Cusabio, Wuhan, China) and diamine oxidase (DAO) (cat.no. CSB-E10090m, Cusabio, Wuhan, China) were measured with ELISA kits according to the manufacturer’s protocol to evaluate the gut permeability. Since lactic acid is a small molecule lacking antigenic epitopes, we use a colorimetric assay kit (cat.no. E-BC-K002-M, Elabscience, Wuhan, China) to detect its serum concentration.

### 2.4. Sequencing of 16s rRNA and Bioinformatics

Four weeks after ligation, fecal samples were collected in sterile freezing tubes and immediately stored at −80 °C under sterile conditions [26]. After microbial DNA from fecal samples was extracted via the E.Z.N.A.^®^ soil DNA Kit (Omega Bio-tek, Norcross, GA, USA), the quality and concentration of the DNA were determined using 1.0% agarose gel electrophoresis and a NanoDrop^®^ ND-2000 spectrophotometer (Thermo Scientific Inc., Waltham, MA, USA), respectively. The amplification of V3–V4 region in the bacterial 16S rRNA gene was carried out with primer pairs 338F (5′-ACTCCTACGGGAGGCAGCAG-3′) and 806R (5′-GGACTACHVGGGTWTCTAAT-3′) [27] by an ABI GeneAmp^®^ 9700 PCR thermocycler (ABI, Foster City, CA, USA). The purified amplicons were combined in equal proportions and subjected to paired-end sequencing using an Illumina MiSeq PE300 platform (Illumina, San Diego, CA, USA) following the standard protocols by Majorbio Bio-Pharm Technology Co. Ltd. (Shanghai, China). Next, we grouped the sequences that had been optimized into operational taxonomic units (OTUs) using UPARSE 7.1 [28]. This clustering process was based on a similarity threshold of 97% in sequence similarity level.

Bioinformatic analysis of the gut microbiota was carried out using the Majorbio Cloud platform (https://cloud.majorbio.com, accessed on 20 February 2025) and R software (version 4.2.2). Based on the OTU information, α-diversity indexes were calculated with Mothur v1.30.1 [29], and β-diversity was determined by non-metric multidimensional scaling (NMDS) based on unweighted unifrac and Bray–Curtis dissimilarity using the Vegan v2.5-3 package. Linear discriminant analysis (LDA) effect size (LEfSe) [30] was performed to identify the significantly abundant taxa (from phylum to genera) of bacteria among the different groups (LDA score > 3, *p* < 0.05). Co-occurrence networks were created to investigate how different parts of the community interacted within the samples [31]. A correlation between two nodes was considered to be statistically robust if Spearman’s correlation coefficient was greater than 0.8 or less than −0.8, with a corresponding *p*-value of less than 0.05. To predict microbial functions, PICRUSt2 analysis [32] was performed based on OTU representative sequences. Procrustes analysis, redundancy analysis (RDA), and distance-based redundancy analysis (db-RDA) were applied to confirm the association between major clinical parameters and gut microbiota [33]. Spearman correlations were also conducted to investigate the relationship between gut microbiota and intestinal barrier indexes.

### 2.5. Statistical Analysis

Statistical analysis was performed with GraphPad Prism 8.0 software or R software (version 4.2.2). Gaussian distribution of the data was tested using the Shapiro–Wilk test, and Levene’s test was utilized to assess the homogeneity of variances. The significance of differences among the four experimental groups was analyzed using one-way ANOVA for normally distributed data and the Kruskal–Wallis test for non-normally distributed data. We used Student’s *t*-test or the Wilcoxon rank-sum test as appropriate, and *p*-values were adjusted using the Benjamini–Hochberg method. All data were presented as the mean ± standard deviation (SD), and *p* < 0.05 was considered statistically significant.

## 3. Results

### 3.1. HGF May Increase Intestinal Permeability and Escalate Gut Barrier Disorder During the Periodontitis Period

**Figure 1:**
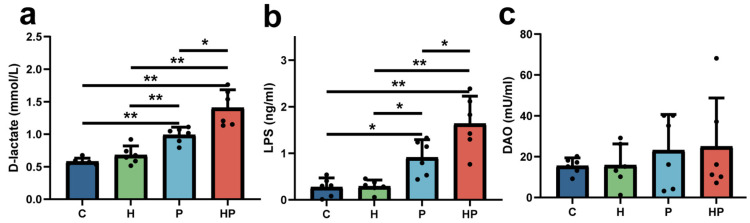
Representation of intestinal permeability. Serum level of D-lactate (**a**), LPS (**b**), and DAO (**c**) in the C, H, P, and HP groups (n = 6 per group). C: control group; H: HGF-Tg mice; P: WT mice with periodontitis; HP: HGF-Tg group with periodontitis; *, *p* < 0.05; **, *p* < 0.01. One-way ANOVA and Student’s *t*-test were used.

The serum levels of LPS and D-lactate were elevated in both the WT and HGF-Tg mice with periodontitis compared with the controls. More importantly, the concentrations of serum LPS and D-lactate were significantly higher in the HGF-Tg mice than that in the WT mice during periodontitis (Figure 1a,b). Although the serum level of DAO slightly rose in the P and HP groups in comparison to controls, there was no significant difference among them (Figure 1c).

**Figure 2:**
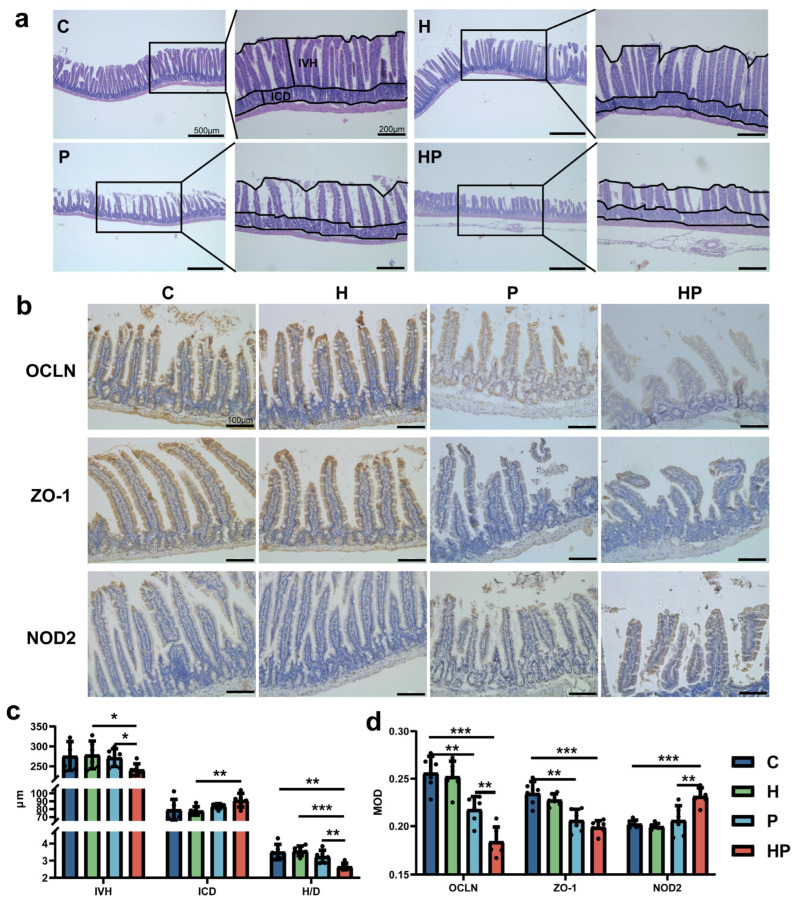
Representation of mechanical gut barrier and immune gut barrier: (**a**) representative HE staining images displaying ileum across the four groups (scale bar, 500 μm or 200 μm); (**b**) representative immunohistochemistry staining of ileum for OCLN (Occludin), ZO-1, and NOD2 (scale bar, 100 μm); (**c**) assessment of IVH, ICD, and H/D (n = 6 per group); (**d**) mean density (MOD) of OCLN, ZO-1, and NOD2 (n = 6 per group). *, *p* < 0.05; **, *p* < 0.01; ***, *p* < 0.001. One-way ANOVA and Student’s *t*-test were used.

The gut mechanical barrier was analyzed by IVH, ICD, and H/D in intestinal villi. The HP group exhibited reduced IVH and elevated ICD, together with lower H/D (Figure 2a,c). Immunohistochemical staining showed the intestinal level of occludin and ZO-1 (key components of epithelial tight junctions critical for maintaining intestinal barrier integrity), which were reduced in the P group in contrast to the controls. In addition, HGF further significantly facilitated the downregulation of occludin in the context of periodontitis (Figure 2b,d). Nevertheless, there existed no significant difference between the P group and the HP group regarding ZO-1 levels (Figure 2b,d).

In addition to the mechanical barrier, immune-associated proteins such as NOD2 are essential components affecting gut barrier and permeability. In our study, the intestinal level of NOD2 was higher in the HP group than that in the other three groups (Figure 2b,d).

### 3.2. HGF Altered Gut Microbiota Diversity in Mice

**Figure 3:**
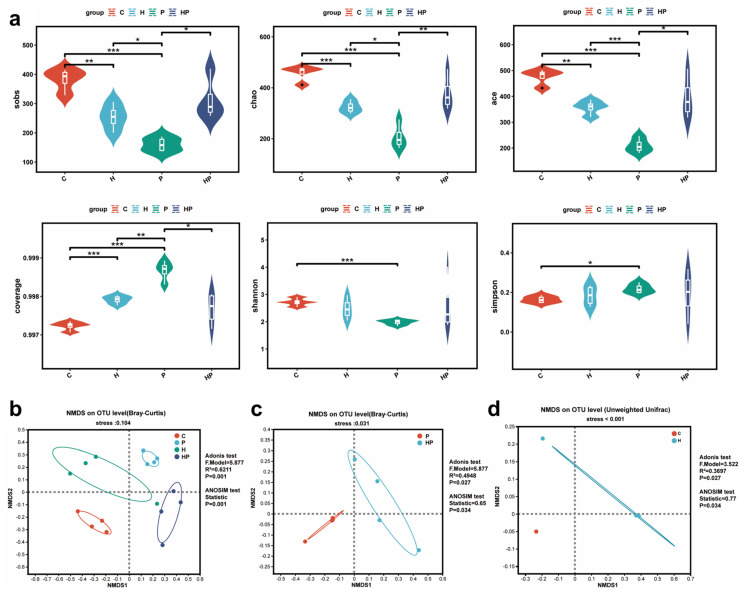
α-Diversity and β-diversity of gut microbiota: (**a**) gut microbiota α-diversity was measured using sobs, chao1, ace, coverage, Shannon, and Simpson indexes; (**b**–**d**) sample ordination was shown by NMDS. *, *p* < 0.05; **, *p* < 0.01; ***, *p* < 0.001.

We calculated α-diversity indexes including sobs, chao1, ace, coverage, Shannon, and Simpson, revealing significant differences in the gut microbiota among the four groups. Periodontitis altered all mentioned α-diversity indexes, and the sobs, chao1, and ace indexes were significantly higher in HP group compared with the P group (Figure 3a). However, there was no significant discrepancy in the Shannon and simpson indexes between the HP group and the P group (Figure 3a). Furthermore, the β-diversity analysis revealed distinct segregation of intestinal microbiota within each group, indicating substantial variations in the gut microbiota composition among the different experimental groups (R^2^ = 0.6211, *p* = 0.001) (Figure 3b). The stress values of NMDS, all below 0.2, demonstrated a strong correspondence between the samples and groups in multidimensional space (Figure 3b). Interestingly, the NMDS plot also illustrated that the microbial communities in the HGF-Tg mice clustered separately from those in the WT mice whether or not periodontitis was present (Figure 3c,d).

### 3.3. HGF Modulated Intestinal Microbiological Compositions When Periodontitis Occurred

**Figure 4:**
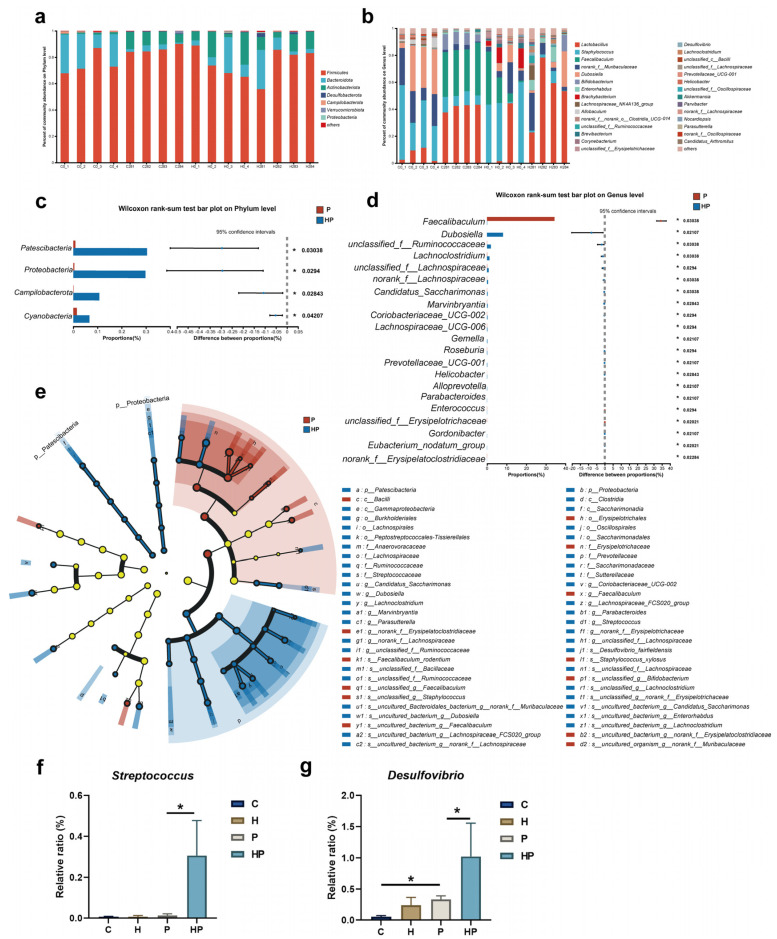
Proportions in gut microbiota profile: (**a**,**b**) proportions of gut bacterial groups at the phylum level and genus level among the four groups; (**c**,**d**) comparison of proportions and differences between proportions at the phylum level and genus level in the P and HP groups; (**e**) LEfSe analysis identified taxa with differential abundance, characterized by an LDA score exceeding 3.0 and a significance level of *p* < 0.05; (**f**) comparison of proportion of *Streptococcus* among the four groups; (**g**) comparison of the proportion of *Desulfovibrio* among the four groups C, WT mice; H, HGF-Tg mice; 0, non-periodontitis group (teeth ligation for 0 day); 28, periodontitis group (teeth ligation for 28 days); *, *p* < 0.05; Kruskal–Wallis test and Wilcoxon rank-sum test were used.

To delve into the impact of periodontitis and HGF on the composition of gut microbiota, we analyzed the abundance of gut microbiota in all samples (Figure 4a,b). Through the Kruskal–Wallis test, our results demonstrated different abundances of gut bacterial groups at the phylum and genus level among the four groups (Figure S2a,b). Our results attested that the enrichment of *Patescibacteria*, *Proteobacteria*, *Campilobacterota*, and *Cyanobacteria* was responsible for differences in abundance at the phylum level in HGF-Tg mice with periodontitis (Figure 4c). We also conducted an LEfSe analysis, with an LDA score threshold of 3.0, to identify variations in gut taxa between the P and HP groups. At the genus level, we found that the proportions of *Dubosiella*, *g_unclassified_f_Ruminococcaceae*, *Lachnoclostridium*, *Lachnospiraceae_UCG-006*, *Candidatus_Saccharimonas*, *Marvinbryantia*, *Coriobacteriaceae_UCG-002*, *Gemella*, *Roseburia, Prevotellaceae_UCG-001*, *Helicobacter*, *Alloprevotella*, *Parabacteroides*, *Gordonibacter*, *Eubacterium_nodatum_group*, and *Mucispirillum* were significantly elevated in the HP group, while the abundances of *Faecalibaculum*, *Enterococcus*, *g_unclassified_f_Erysipelotrichaceae*, and g_*norank_f_Erysipelatoclostridiaceae* were profoundly lower in the HP group than in the P group (Figure 4d,e). Notably, the abundance of periodontitis-associated pathogens, including *Streptococcus* and *Desulfovibrio,* increased in the HP group compared to the P group (Figure 4f,g). We also observed that HGF significantly altered the abundance of some bacterial taxa in mice without periodontitis, suggesting HGF still plays a momentous role in the gut microbiota even in the absence of periodontitis (Figure S2c–e).

### 3.4. HGF Facilitated Different Co-Occurrence Network of Gut Bacterial Taxa

**Figure 5:**
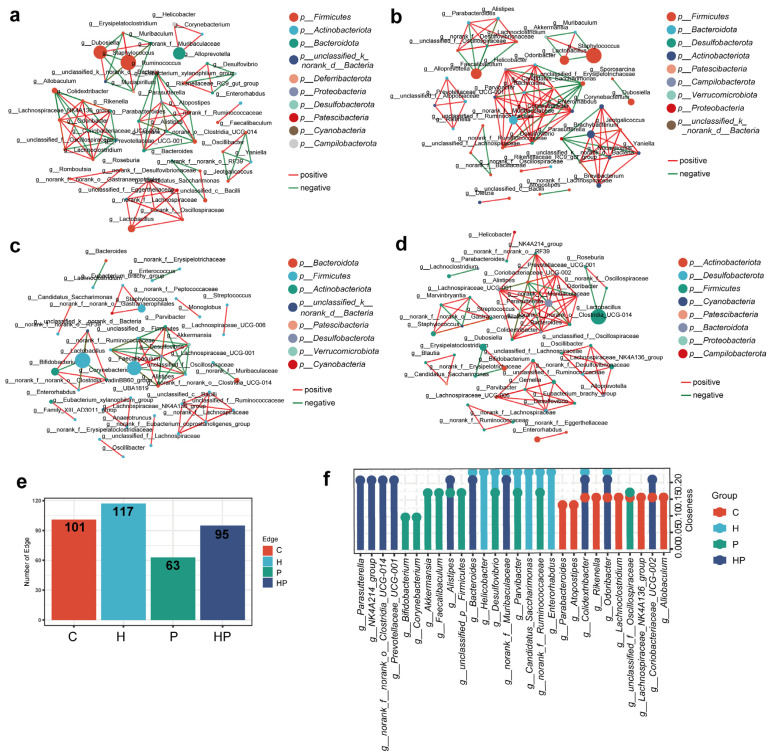
Correlation network of gut microbiota of different groups. A network analysis of the top 50 abundant genera with a Spearman correlation coefficient >0.8 or <−0.8 in the C group (**a**), H group (**b**), P group (**c**), and HP group (**d**). Each node within the network represents a bacterial genus, with circle size denoting relative abundance. The color of circles indicates their phylum-level classification. The color of codes signifies positive (red) and negative (green) correlations. Significance levels were set at *p* < 0.05. (**e**) Edges in four groups. (**f**) Closeness centralities among nodes in different groups.

To infer potential interactions among the taxa of the gut bacterial community in different groups, we performed a co-occurrence network of the bacterial genus using Spearman’s correlation. We visualized strong correlations (*p* < 0.05, correlation coefficient > 0.8 or <−0.8) in this analysis. Our findings showed that the correlation network in the P group was simpler and had fewer edges than that in the C and H groups, indicating that periodontitis affected the interaction relationship of the intestinal microbiota (Figure 5a–e). However, the correlation network in the HP group was more complex than that in the P group, with different components and more edges (Figure 5a–e).

Subsequently, we computed closeness centrality, a measure of node importance within bacterial networks, to estimate the structural importance of individual bacterial taxa within each network. A lollipop chart manifested the top 10 closeness centralities of microorganisms (Figure 5f). Pro-inflammatory bacteria including *g_Prevotellaceae_UCG-001*, *g_Bacteroides*, and *g_NK4A214_group* inhibited high closeness in the HP group, indicating that these pro-inflammatory genera may act as the core microbiome that interacts with other species to play an important role in gut barrier injury.

### 3.5. HGF Transformed Microbial Functions in Mice with Periodontitis

**Figure 6:**
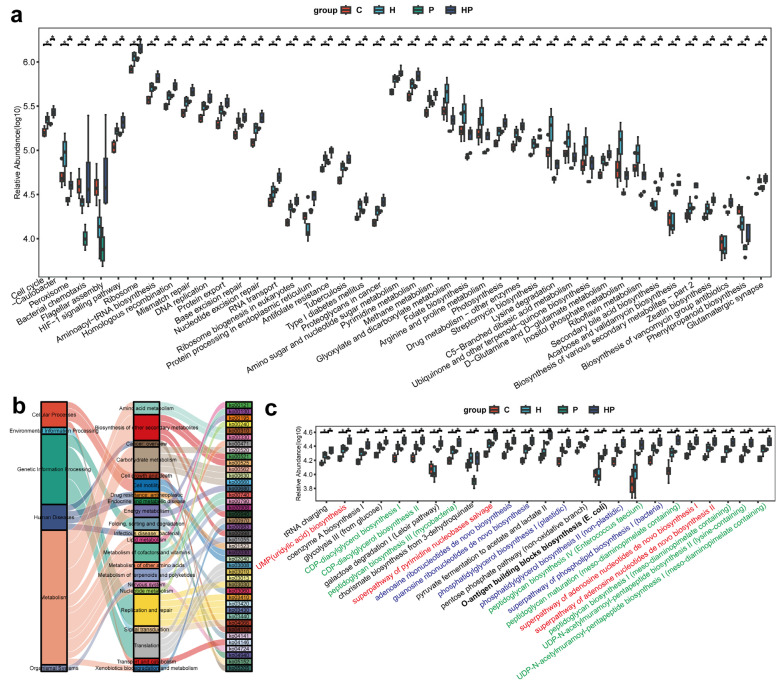
Differential PICRUSt-predicted biological pathways: (**a**) the 42 differential KEGG pathways were classified; (**b**) the Sankey plot illustrates categories of different KEGG levels; (**c**) the predicted gut metabolism pathways based on MetaCyc. The Kruskal–Wallis test and Wilcoxon rank-sum test were used to determine statistically discrepancy. *, *p* < 0.05.

To investigate the differential biological pathways in the four groups, we utilized PICRUSt to predict them based on the 16S rRNA gene sequencing data. We identified 42 differential KEGG pathways, which were classified based on their average relative abundance values (log10) (Figure 6a). The differential KEGG pathways were then visualized using a Sankey plot (Figure 6b), revealing that the majority of the differential pathways were metabolic pathways, including biosynthesis of other secondary metabolites, carbohydrate metabolism, amino acid metabolism, metabolism of terpenoid and polyketides, and so on. To further elucidate the impact of HGF on metabolism, we analyzed the metabolic pathways using the MetaCyc (Metabolic Pathways From all Domains of Life) database (Figure 6c). HGF further activated the biosynthesis of peptidoglycan and CDP-diacylglycerol, which are indispensable for bacterial cell walls. In addition, the biosynthesis of nucleotides, the basic structure of genetic material, was remarkably enriched in the HP group compared with the P group. Moreover, the abundance of o-antigen building block biosynthesis was highly elevated in the HP group, implying that the gut bacteria of the HP group were inclined to produce LPS and thereby cause intestinal damage. Our results indicated that HGF potentially affects bacterial proliferation and virulence factor synthesis, which may contribute to a worsened condition to some extent.

### 3.6. Identification of Key Gut Microbiota Associated with Intestinal Barrier

**Figure 7:**
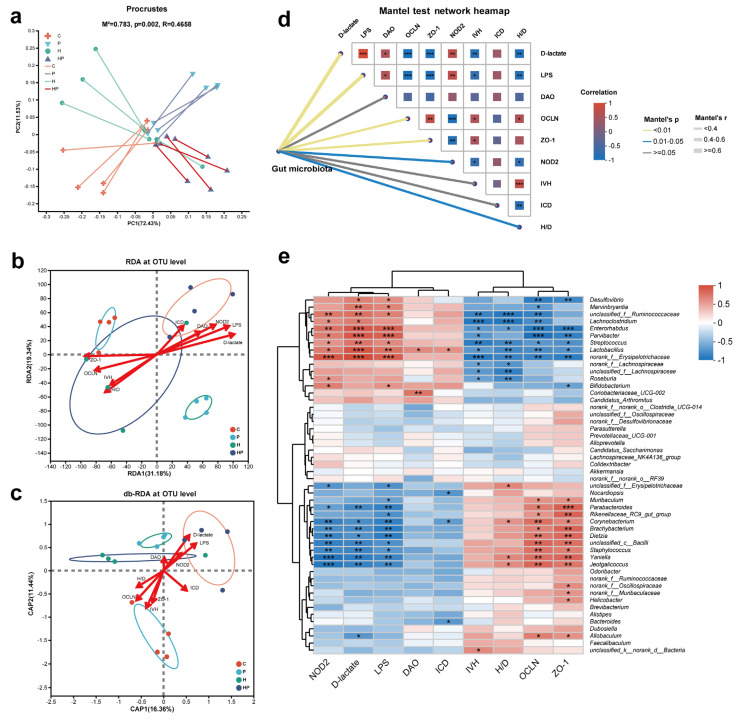
Association between intestinal barrier indexes and gut microbiota: (**a**) Procrustes analysis demonstrated association between microbial community and environmental factors; (**b**,**c**) RDA and db-RDA of bacterial diversity and intestinal barrier indexes among four groups; (**d**) Mantel test network heatmap; (**e**) Spearman correlation between top 50 gut microbial genera and gut barrier measurements, with significance levels marked as *, *p* < 0.05; **, *p* < 0.01; and ***, *p* < 0.001.

Given the association between the gut microbiota and intestinal barrier, we performed a Procrustes analysis, preliminarily revealing the consistency between gut barrier changes and microbial alterations (Figure 7a). To further investigate the effects of gut barrier indexes on intestinal microbiota changes, RDA was utilized to evaluate the relationships between these features and gut microbiota at the OTU level (Figure 7b). Our results indicated that D-lactate, LPS, OCLN, ZO-1, NOD2, and H/D significantly influenced the alteration of gut microbiota (Table 1). Additionally, D-lactate, LPS, and NOD2 mainly positively contributed to gut microbiota distribution in the P group and HP group, while OCLN, ZO-1, and H/D were primarily positively responsible for microbial composition in the C group and H group. The magnitude of the impact of D-lactate, LPS, and NOD2 on taxa was higher in the HP group compared to the P group. However, the RDA reflects the Euclidean distance between the quadrat on the sorting diagram, which does not exactly fit the characteristics of a microbial community. Consequently, we conducted a db-RDA and found that only D-lactate and LPS were notably positively associated with the gut microbiota in P group and HP group (Figure 7c, Table 2), in keeping with the result of Mantel test (Figure 7d).

Subsequently, we explored the correlation between the characteristics above and gut taxa at the genus level (top 50 relative abundances of the genera are shown) (Figure 7e). The gut barrier indexes presented two clusters. For instance, *unclassified_f_Ruminococcaceae*, *Streptococcus*, and *Desulfovibrio*, negatively associated with OCLN, exhibited a positive relationship with the serum level of D-lactate and LPS. In contrast, *Brachybacterium* and *Corynebacterium*, positively correlated with OCLN and ZO-1, presented a negative association with D-lactate, LPS, and NOD2

## 4. Discussion

The systemic consequences of periodontitis via the oral–gut axis have garnered increasing attention, yet two critical gaps persist: (1) the role of host-derived mediators in bridging oral inflammation to gut pathology remains underexplored, and (2) the dual effects of HGF, a regulator of periodontal destruction, on intestinal homeostasis are paradoxically unresolved. Our study bridges these gaps by demonstrating that HGF exacerbates periodontitis-induced gut barrier dysfunction through microbiota–immune crosstalk, thereby positioning HGF as a novel systemic amplifier of oral–gut axis dysregulation.

The gut barrier is generally composed of a mechanical barrier, an immune barrier, and a microbial barrier [2]. The compromised intestinal mechanical barrier in HGF-Tg mice, marked by reduced occludin expression and villous atrophy, underscores HGF’s context-dependent regulation of tight junction (TJ) proteins. While HGF is reported to enhance TJ integrity in isolated intestinal repair models [34], our findings revealed a paradoxical disruptive role in the gut milieu when periodontitis occurred. This dichotomy aligns with emerging evidence that HGF’s effects on TJ proteins are highly dependent on cellular context and disease state. In human breast cancer cells, HGF downregulates TJ molecules (e.g., claudin-1) to promote epithelial–mesenchymal transition [35], while in endothelial monolayers, it reduces transendothelial resistance by inducing tyrosine phosphorylation of occludin and ZO-1, a post-translational modification known to weaken TJ stability [36]. Notably, we observed the selective downregulation of occludin but not ZO-1 in HGF-Tg mice, suggesting that HGF may preferentially target specific TJ components during periodontitis. This selectivity mirrors findings in gastric epithelial cells, where HGF stimulates migration by dissociating ZO-1–occludin complexes without altering total ZO-1 levels [37]. The above evidence preliminary elucidated that HGF may exacerbate the gut mechanical barrier in the context of periodontitis.

Beyond mechanical disruption, HGF profoundly influenced the intestinal immune barrier, particularly through NOD2 upregulation. Mainly expressed in epithelium, NOD2 is a pattern recognition receptor, which can detect bacterial peptidoglycan fragments and other danger signals [38]. Disruption in bacterial communities may trigger NOD2 activation, which in turn regulates MAPK and NF-κB signaling, initiating adaptive immunity and optimizing inflammation [38]. Though the inflammatory and immune response can protect a host against pernicious bacteria, it may also contribute to self-damage [39], including impairing the gut mechanical barrier. Our results demonstrated that HGF further upregulated the intestinal expression of NOD2 to alter the gut immune barrier when periodontitis occurred. Despite that HGF can remise the immune and inflammatory process to protect the epithelium [19], M. Stakenborg et al. demonstrated that HGF could modulate the immune response of neutrophil and Th17 to aggravate colitis, revealing that HGF may intervene in the intestinal immune barrier [20].

Regarding the microbial barrier, the 16S rRNA sequencing showed that HGF contributed to a discrepancy in the intestinal microbiota based on α-diversity and β-diversity whether or not periodontitis was present. This result suggested that HGF may intrinsically modulate gut microbial ecology, independent of periodontal inflammation. Despite that some probiotics such as *Dubosiella* and *Lachnospiraceae_UCG-006* [40,41] were observed as abundant in HGF-Tg mice with periodontitis, we found that the pro-inflammatory bacteria *g_Streptococcus*, *g_unclassified_f_Ruminococcaceae*, *Prevotellaceae_UCG-001*, and *Desulfovibrio_fairfieldensis* [42,43,44,45] were upregulated by HGF when periodontitis occurred. Our correlation analysis also revealed that *g_Streptococcus* and *g_Desulfovibrio* may be involved in impaired gut barrier and immune response, consistent with previous studies [42,43,44,45]. Interestingly, *g_Streptococcus* and *g_Desulfovibrio* are important components of periodontitis pathogens [10,46]. Studies have shown that periodontitis can promote the translocation of these pathogens in the gut, thereby causing intestinal barrier disruption and systemic injury [10,46,47]. These results indicated that HGF may act as a host factor to exacerbate this microbial-translocation-induced gut mechanical and immune barrier dysfunction. Meanwhile, HGF diminished the enrichment of *Faecalibaculum* and *g_norank_f__Erysipelatoclostridiaceae* in the context of periodontitis, which are associated with anti-inflammatory properties [48,49]. For instance, *Faecalibaculum* can modulate retinoic acid signaling to maintain intestinal epithelial homeostasis [50]. The dynamic relationship among the gut microbiota also plays a crucial role in the integrity of the microbial barrier and immune barrier [51]. Therefore, we conducted a co-occurrence network analysis of bacterial taxa, revealing that periodontitis led to apparently simpler microbial interactions, whereas HGF promoted a more complex correlation network when periodontitis was present, consistent with the aforementioned result of α-diversity. Although some studies have suggested that a complex network of microbiota provides a more stable microbial barrier to protect gut integrity [51], our result illustrated that some pro-inflammatory members including *g_Prevotellaceae_UCG-001*, *g_Bacteroides*, *Alistipes*, and *g_NK4A214_group* [42,43,52] acted as core microbiome components interacting with other species, thus favoring gut barrier damage. Additionally, the PICRUSt2 analysis depicted distinctly contrasting bacterial functions altered by HGF when periodontitis occurred, particularly in metabolic functions. Consequently, we identified divergence in metabolic pathways using the MetaCyc database, suggesting that HGF may enhance bacteria proliferation by upregulating the biosynthesis of peptidoglycan phospholipid and nucleotides [53]. Furthermore, the pathway involved in o-antigen biosynthesis, an essential component of LPS, was more highly enriched in the HGF-Tg mice with periodontitis, which aligned with the elevated serum level of LPS. In a comprehensive perspective, HGF further disrupted the structure and function of the gut microbiome, ultimately leading to gut damage in spite of an increase in some probiotics.

The convergence of mechanical, immune, and microbial barrier disruptions in HGF-overexpressing mice culminates in a “leaky gut” phenotype—a hallmark of oral–gut axis dysregulation [54]. Elevated serum levels of D-lactate, LPS, and DAO in HGF-Tg mice with periodontitis collectively demonstrated that HGF exacerbated intestinal hyperpermeability, enabling microbial products to translocate systemically. This aligns with the emerging paradigm wherein periodontitis acts as a “remote driver”, not only through direct gut microbial dissemination [55] but also via host-derived mediators like HGF that amplify gut barrier fragility. While our study focused on intestinal endpoints, this leaky gut phenotype provides a plausible mechanistic bridge between periodontitis and extraoral comorbidities [56,57]. For instance, circulating LPS from both oral and gut sources is implicated in metabolic endotoxemia driving insulin resistance [56], and DAO-mediated histamine release may exacerbate allergic responses [57].

Despite these advances, the systemic ramifications of HGF-modulated gut leakage remain speculative in our model. Future studies should assess direct endpoints like hepatic inflammation or adipose tissue dysfunction to confirm whether oral–gut axis disruption via HGF truly propagates systemic disease. This limitation underscores the need for multi-organ profiling in periodontitis models to fully unravel the oral–gut–systemic triad. Additionally, as our study employed a transgenic model with systemic HGF overexpression, the local effects of exogenous HGF remain to be verified. Future studies using local HGF administration in wild-type models are needed to distinguish direct HGF action from secondary systemic influences. In addition, mechanistic dissection of HGF-related signaling will further clarify its role in the oral–gut–systemic axis and inform therapeutic strategies.

## 5. Conclusions

**Figure 8:**
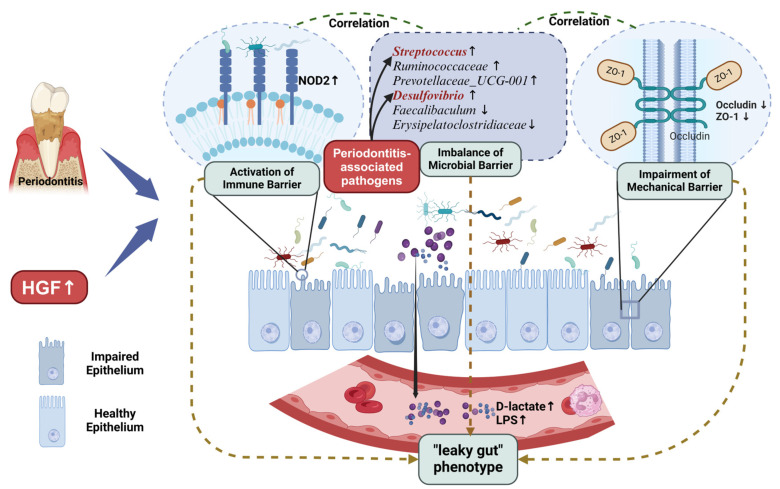
The effects of HGF on gut permeability and intestinal barrier (mechanical barrier, immune barrier, and microbial barrier) in mice with periodontitis. The illustration was created with BioRender (https://www.biorender.com/, accessed on 20 February 2025).

Our study established HGF as a potential host-derived mediator of oral–gut axis dysregulation in periodontitis. By integrating transgenic models with multi-omics approaches, we demonstrate that HGF overexpression exacerbates gut mechanical, immune, and microbial barrier dysfunction. These alterations collectively drive a “leaky gut” phenotype, characterized by elevated systemic LPS and D-lactate—biomarkers linking oral inflammation to distant pathologies (Figure 8). These findings bridge two critical gaps in periodontitis research: the underexplored role of host signaling molecules in oral–gut crosstalk and the paradoxical duality of HGF in gut homeostasis. While current therapeutic strategies focus on microbial control, our work suggests that targeting HGF or its modulated microbial consortia (*Faecalibaculum* supplementation) may synergistically mitigate both oral and systemic complications. Future studies should validate causality through fecal transplantation and delineate how HGF-driven gut leakage propagates multi-organ dysfunction, ultimately advancing precision interventions for periodontitis-associated comorbidities.

## Figures and Tables

**Table 1:** The RDA analysis between gut microbiota and intestinal barrier indicators. *, *p* < 0.05; **, *p* < 0.01.

	RDA1	RDA2	R^2^	*p* Value
D-lactate	0.9698	0.244	0.7149	0.001 **
LPS	0.933	0.3598	0.6751	0.005 **
DAO	0.8887	0.4586	0.2643	0.118
OCLN	−0.9715	−0.2371	0.4466	0.028 *
ZO-1	−0.9999	−0.0116	0.5769	0.003 **
NOD2	0.8939	0.4482	0.4821	0.02 *
IVH	−0.8418	−0.5398	0.3366	0.079
ICD	0.6777	0.7354	0.1712	0.299
H/D	−0.8107	−0.5855	0.4562	0.027 *

**Table 2:** The db-RDA analysis between gut microbiota and intestinal barrier indicators. *, *p* < 0.05.

	CAP1	CAP2	R^2^	*p* Value
D-lactate	0.6531	0.7573	0.3892	0.035 *
LPS	0.8435	0.5371	0.3842	0.039 *
DAO	−0.0091	1	0.0267	0.857
OCLN	−0.7471	−0.6647	0.3335	0.063
ZO-1	−0.3224	−0.9466	0.2213	0.19
NOD2	0.7889	0.6145	0.1223	0.45
IVH	−0.4423	−0.8969	0.3318	0.094
ICD	0.8073	−0.5902	0.2308	0.188
H/D	−0.9128	−0.4084	0.2126	0.223

## Data Availability

The raw 16s rRNA sequencing data have been submitted to NCBI under the SRA database [PRJNA1019142].

## Supplementary Materials

The following supporting information can be downloaded at https://www.mdpi.com/article/10.3390/biology14050496/s1, Figure S1: The DNA electrophoresis analysis revealed distinct bands corresponding to the amplified DNA fragments of HGF in intestines from HGF-Tg mice; Figure S2: Identification of different microbial biomarkers.

## Abbreviations

HGF	Hepatocyte Growth Factor
HGF-Tg	Hepatocyte Growth Factor High-Expression Transgenic
LPS	Lipopolysaccharide
DAO	Diamine Oxidase
ZO-1	Zonula Occludens-1
NOD2	Nucleotide-binding Oligomerization Domain-containing Protein 2
c-Met	Cellular-Mesenchymal Epithelial Transition Factor
WT	Wild Type
C	WT Mice
H	HGF-Tg Mice
P	WT Mice with Periodontitis
HP	HGF-Tg Mice with Periodontitis
HE	Hematoxylin and Eosin
IVH	Intestinal Villus Height
ICD	Intestinal Crypt Depth
H/D	Ratio of Villus Height to Crypt Depth
MOD	Mean Density
IOD	Integrated Density
OTU	Operational Taxonomic Units
NMDS	Non-metric Multidimensional Scaling
LDA	Linear Discriminant Analysis
LEfSe	Linear Discriminant Analysis Effect Size
PICRUSt	Phylogenetic Investigation of Communities by Reconstruction of Unobserved States
RDA	Redundancy Analysis
db-RDA	Distance-based Redundancy Analysis
MetaCyc	Metabolic Pathways From all Domains of Life
TJ	Tight junction
