# Supplementary material for: HGF Aggravated Periodontitis-Associated Gut Barrier and Microbial Dysfunction: Implications for Oral–Gut Axis Regulation
Source: Biology (Basel). 2025 May 2;14(5):496. doi: 10.3390/biology14050496 (PMC12109049; doi:10.3390/biology14050496)
Supplement: Supplementary file 1 [file biology-14-00496-s001.zip › biology-3555886-supplementary.pdf]

HGF Aggravated Periodontitis-associated Gut Barrier and Microbial Dysfunction: Implications for Oral-Gut Axis Regulation

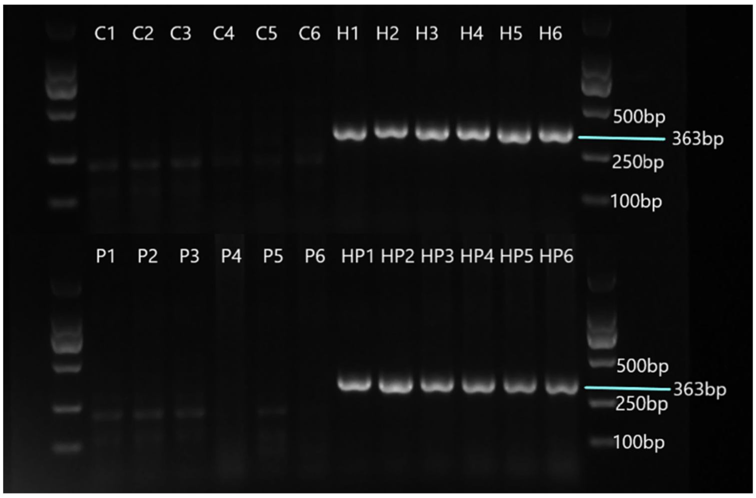

Figure S1. The DNA electrophoresis analysis revealed distinct bands corresponding to the amplified DNA fragments of HGF in intestine from HGF-Tg mice. C, Wt mice; H, HGF-Tg mice; P, Wt mice with periodontitis; HP, HGF-Tg mice with periodontitis

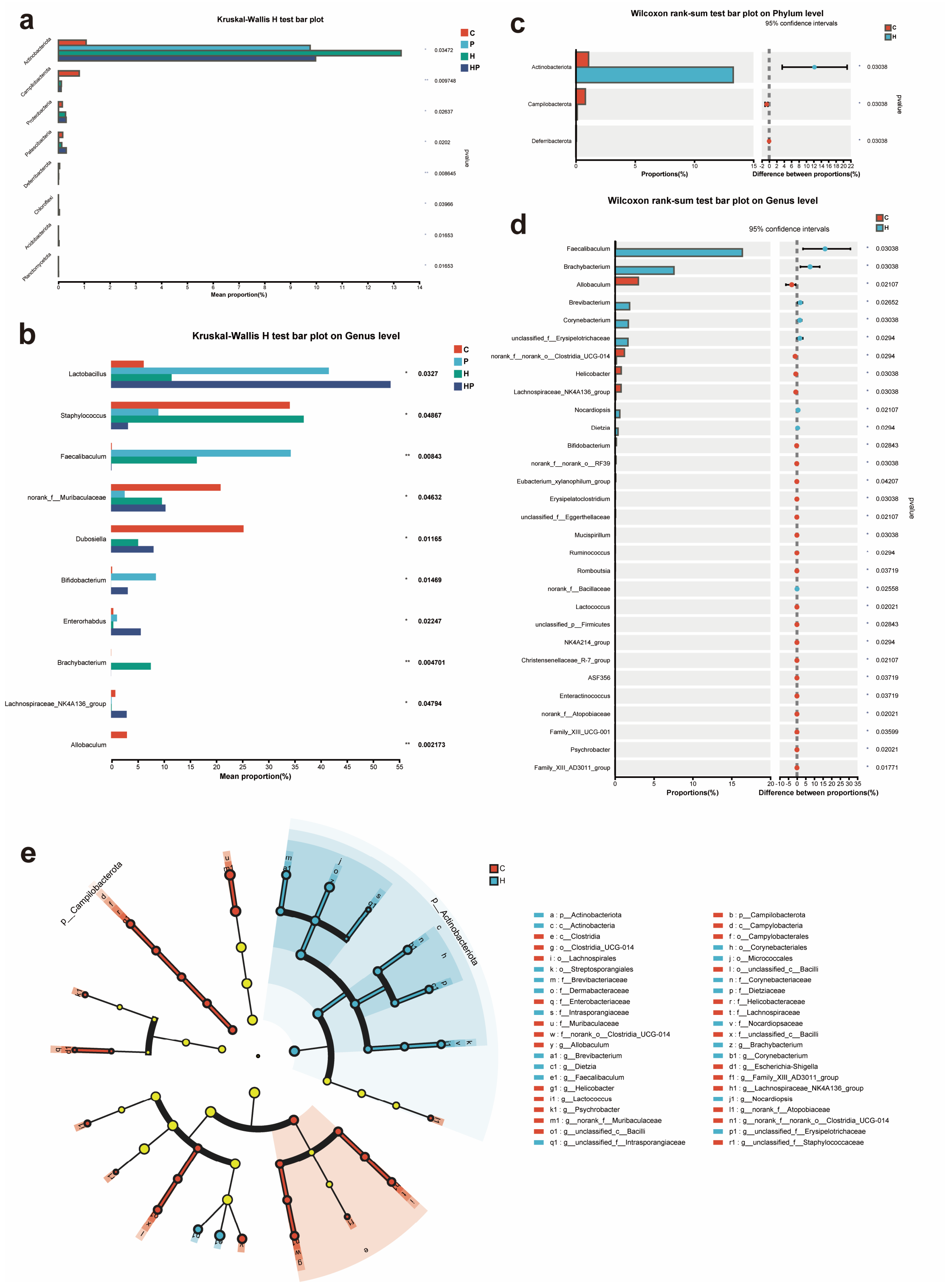

Figure S2. Identification of different microbial biomarkers. (a, b) Comparison of proportions and difference between proportions at phylum level and genus level among four groups. (c, d) Comparison of proportions and difference between proportions at phylum level and genus level between the C group and the H group. (e) Linear discriminant analysis (LDA) revealed differentially abundant taxa as biomarkers ($P < 0.05$) with LDA score > 3.0
